# Biological Effects of Anti-RANKL Antibody and Zoledronic Acid on Growth and Tooth Eruption in Growing Mice

**DOI:** 10.1038/s41598-019-56151-1

**Published:** 2019-12-27

**Authors:** Motoki Isawa, Akiko Karakawa, Nobuhiro Sakai, Saki Nishina, Miku Kuritani, Masahiro Chatani, Takako Negishi-Koga, Masashi Sato, Mitsuko Inoue, Yukie Shimada, Masamichi Takami

**Affiliations:** 1 Department of Pediatric Dentistry, Showa University School of Dentistry, 2-1-1 Kitasenzoku, Ota-ku, Tokyo 145-8515 Japan; 2 Department of Pharmacology, Showa University School of Dentistry, 1-5-8 Hatanodai, Shinagawa-ku, Tokyo 142-8555 Japan; 3 Pharmacological Research Center, Showa University, Tokyo, 142-8555 Japan; 4 Department of Special Needs Dentistry for Persons with Disabilities, Showa University School of Dentistry, 2-1-1 Kitasenzoku, Ota-ku, Tokyo 145-8515 Japan; 5 Department of Mucosal Barriology, International Research and Development for Mucosal Vaccines, The Institute of Medical Science, The University of Tokyo, 4-6-1 Shirokanedai, Minato-ku, Tokyo 108-8639 Japan

**Keywords:** Bone development, Paediatric research

## Abstract

The anti-bone resorptive drugs denosumab, an anti-human-RANKL antibody, and zoledronic acid (ZOL), a nitrogen-containing bisphosphonate, have recently been applied for treatment of pediatric patients with bone diseases, though details regarding their effects in growing children have yet to be fully elucidated. In the present study, we administered these anti-resorptive drugs to mice from the age of 1 week and continued once-weekly injections for a total of 7 times. Mice that received the anti-RANKL antibody displayed normal growth and tooth eruption, though osteopetrotic bone volume gain in long and alveolar bones was noted, while there were nearly no osteoclasts and a normal of number osteoblasts observed. In contrast, ZOL significantly delayed body growth, tooth root formation, and tooth eruption, with increased osteoclast and decreased osteoblast numbers. These findings suggest regulation of tooth eruption via osteoblast differentiation by some types of anti-resorptive drugs.

## Introduction

Bone is dynamic tissue, and continued bone modeling during the neonatal and adolescent periods is essential for vertebrate growth. Normal bone development is maintained by a balance between formation by osteoblasts and resorption by osteoclasts^1^, while enhanced bone resorption by osteoclasts can lead to development of bone diseases, such as osteoporosis and bone metastasis^2,3^. Osteoclast differentiation and function are regulated by a key cytokine termed receptor activator of nuclear factor-κB ligand (RANKL)^4^, a type II transmembrane protein and member of the tumor necrosis superfamily that is produced by bone marrow stromal cells, osteocytes, and osteoblasts^4,5^. When RANKL binds to its receptor RANK, monocyte-macrophage progenitors differentiate into osteoclasts and induce bone resorption^4^. Due to their inhibitory effects towards osteoclasts, anti-resorptive drugs such as denosumab and bisphosphonates are used to treat patients with osteoclastic bone disease.

Denosumab, a novel anti-resorptive drug, is a fully human monoclonal anti-RANKL antibody that binds to RANKL, and strongly inhibits osteoclast differentiation and bone resorption^6^. On the other hand, zoledronic acid (ZOL) is a nitrogen-containing bisphosphonate and one of the most potent known inhibitors of bone resorption, with a known affinity for hydroxyapatite^7^. When isolated from bone surfaces by resorption of osteoclasts by bone tissues, ZOL induces cell apoptosis and functional decline via inhibition of mevalonate metabolism^8^. Because of their strong therapeutic effects, denosumab and ZOL are routinely given to adult patients for treatment of bone destruction^9–11^.

In recent years, denosumab and ZOL have also been applied for treatment of bone diseases in child cases, such as osteogenesis imperfecta^12,13^, giant cell bone tumors^14,15^, and juvenile-onset osteoporosis^16,17^. Both can increase bone mineral density^12,13^ and also ameliorate pain associated with bone tumors in children^14,18^. However, there is insufficient information in regard to efficacy and toxicity, thus use of anti-resorptive drugs in pediatric patients remains controversial^19,20^. Child bone diseases are known to inhibit hard tissue development, for example, osteogenesis imperfecta has been shown to evoke growth suppression and dentinogenesis imperfecta^12,13^, though it remains unclear whether the pathogenesis of abnormal growth in affected children is due to anti-resorptive drug administration or the bone disease itself.

Osteoclasts are essential for bone development and tooth eruption after birth^21,22^, while RANKL deficiency initiates osteopetrotic long bone development and tooth eruption failure^23^. Thus, we hypothesized that osteoclast suppression by anti-resorptive drugs inhibits both bone growth and tooth eruption in developing children. To elucidate the effects and toxicity of anti-resorptive drugs when used for long-term treatment in growing child patients, we continuously administered an anti-mouse-RANKL antibody or a bisphosphonate ZOL to young mice throughout the entire growth phase, and then examined the effects on growth, bone development, and tooth eruption. In addition, to investigate the influence on adults treated during childhood, a single administration was given to infant mice and analysis performed.

## Results

### Mice administered anti-RANKL antibody grew normally, while ZOL injection suppressed body growth

Denosumab does not cross-react with mouse RANKL, thus we used a rat anti-mouse RANKL antibody for this study. Initially, the negative isotype control immunoglobulin G (rat IgG, 2.5 mg/kg) group was compared with the saline (control) group to exclude the possibility of an effect of IgG on growth. Both a single injection and long-term administration resulted in no significant differences regarding survival rate, body growth, and tooth eruption (see Supplementary Figs. S1 and S2).

To clarify the effects of anti-resorptive drugs in adults whose treatment was finished in childhood, we performed a single subcutaneous injection of 2.5 mg/kg of the anti-mouse RANKL antibody, 0.08 mg/kg of ZOL (reference dose: RfD-ZOL), 3.0 mg/kg of ZOL (cumulative dose: CD-ZOL), or saline into 1-week-old mice. The survival rates of mice at 8 weeks of age in the saline, anti-RANKL antibody, RfD-ZOL, and CD-ZOL treatment groups were 100%, 75%, 100%, and 88%, respectively. At the age of 8 weeks, mice treated with the anti-RANKL antibody or RfD-ZOL displayed normal growth, whereas the CD-ZOL-treated mice showed significantly suppressed body length and weight (see Supplementary Fig. S3).

**Figure 1:**
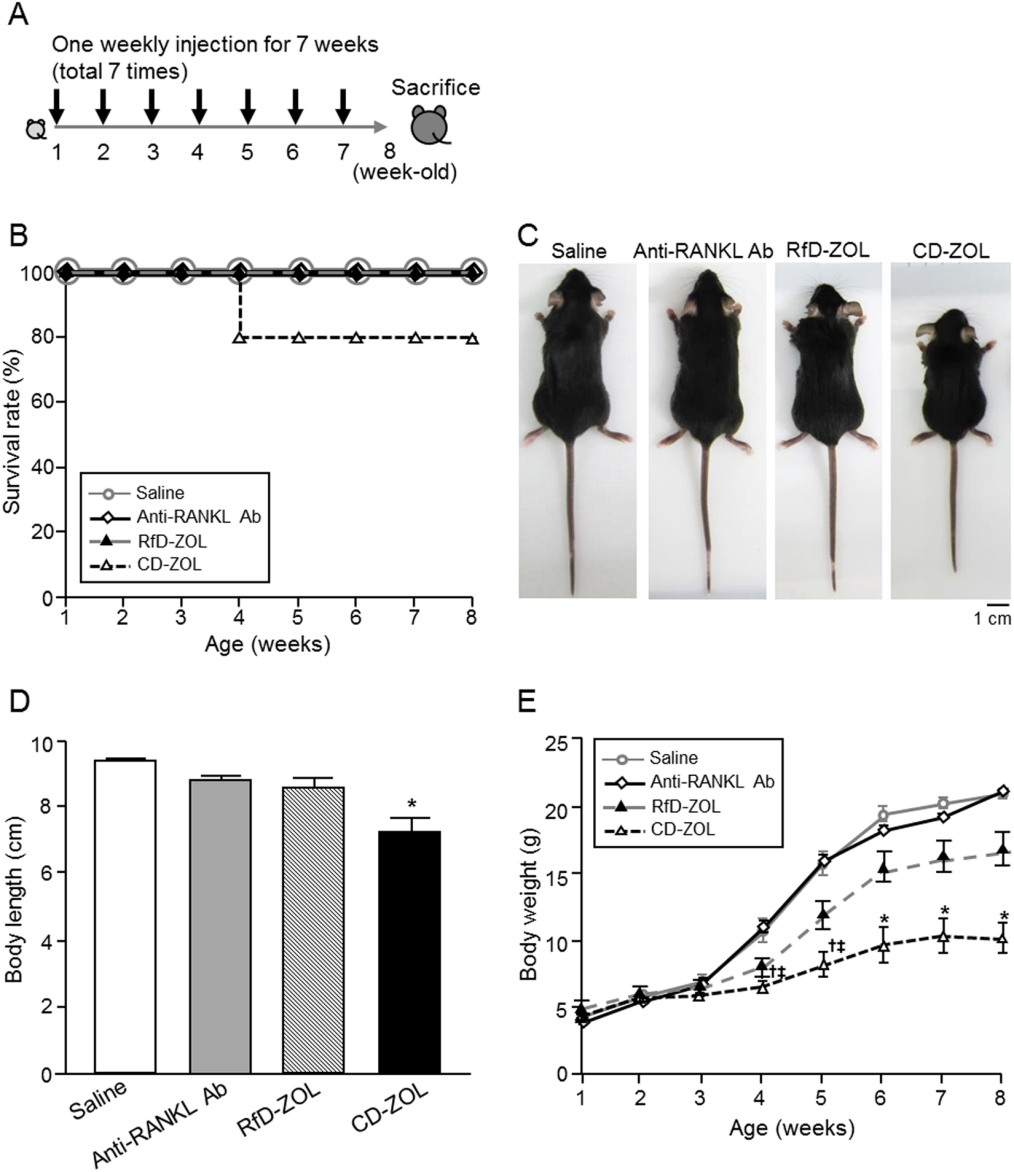
Effects of long-term administrations of anti-RANKL antibody or ZOL on mouse growth. (**A**) Long-term administration protocol. (**B**) Survival rates after once-weekly administration of saline (control, n = 6), anti-RANKL antibody (Ab) at 2.5 mg/kg (n = 5), RfD (reference dose)-ZOL at 0.08 mg/kg (n = 6), or CD (cumulative dose)-ZOL at 3.0 mg/kg (n = 6) for 7 weeks. At 8 weeks of age, the survival rates of mice in those groups were 100%, 100%, 100%, and 83%, respectively. (**C**) Growth appearance and (**D**) naso-anal length of 8-week-old mice after once-weekly administration of saline, anti-RANKL Ab, RfD-ZOL, or CD-ZOL for 7 weeks. (**E**) Weight curves of mice administered saline, anti-RANKL Ab, RfD-ZOL, or CD-ZOL from 1 to 8 weeks of age. There were 6 mice in the saline group, 5 in the anti-RANKL Ab group, 6 in the RfD-ZOL, or 5 in the CD-ZOL group for experiments shown in (**D** and **E**). Statistical differences were assessed by one-way ANOVA with Tukey-Kramer’s test. Statistically significant different from compared to ^*^all the other groups, ^†^saline, or ^‡^anti-RANKL Ab, *p* < 0.05. Error bars represent SEM.

Next, to investigate the long-term effects of anti-resorptive drugs during the growth period, each drug was administered weekly to mice aged 1 to 7 weeks old. The survival rates of mice at 8 weeks of age in the saline, anti-RANKL antibody, RfD-ZOL, and CD-ZOL treatment groups were 100%, 100%, 100%, and 83%, respectively (Fig. 1B). Mice in the anti-RANKL antibody-treated group showed no significant differences regarding naso-anal length and body weight as compared with the saline-injected group. In contrast, body length and weight in the CD-ZOL-treated group were significantly lower as compared to mice in the saline and anti-RANKL antibody groups (Fig. 1C–E). Furthermore, the RfD-ZOL-treated group showed a tendency for growth suppression. The growth reduction in the ZOL-treated group occurred in a concentration-dependent manner. The ratio of spleen/total body weight was not significantly different among all of the experimental groups (see Supplementary Fig. S4). All mice showed normal eating behavior throughout the experimental period.

### Anti-RANKL antibody increased femur bone mass and suppressed tibia osteoclastogenesis, while tibia osteoclast number was not significantly suppressed by ZOL

**Figure 2:**
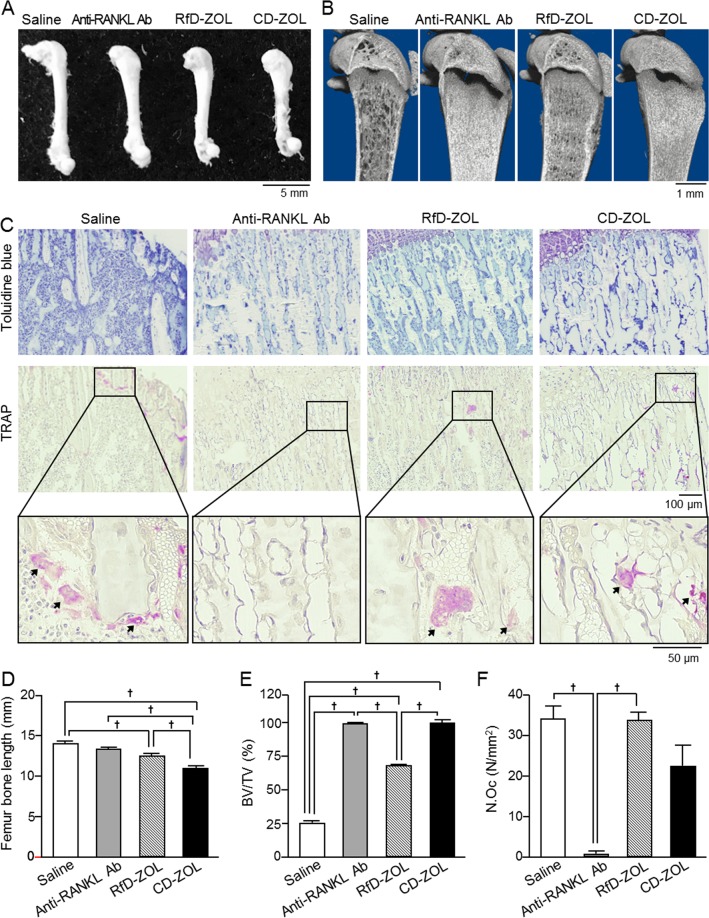
Effects of long-term administrations of anti-RANKL antibody or ZOL on femur and tibia. (**A**) Whole femur appearance. (**B**) Micro-computed tomography (μCT) findings of distal femurs in 8-week-old mice administered saline, anti-RANKL antibody (Ab) at 2.5 mg/kg, RfD-ZOL at 0.08 mg/kg, or CD-ZOL at 3.0 mg/kg weekly for 7 weeks. (**C**) Findings following toluidine blue staining (upper) and TRAP staining (middle and lower) of proximal tibiae growth plate obtained from 8-week-old mice after weekly administrations of each drug for 7 weeks. Higher magnification images of black-boxed regions in middle panels are shown in lower panels. Arrows indicate TRAP-positive cells. Representative results are shown in (**A**–**C**). (**D**) Femur bone length. (**E**) Bone volume/tissue volume (BV/TV) ratios were determined by μCT (saline, n = 6; anti-RANKL Ab, n = 5; Rfd-ZOL, n = 6, CD-ZOL, n = 5). (**F**) Number of osteoclasts (N.Oc) was determined in proximal tibiae following TRAP staining. Four mice from each of the saline, anti-RANKL Ab, RfD-ZOL, and CD-ZOL groups were used. Statistical differences were assessed by one-way ANOVA with Tukey-Kramer’s test. ^†^Statistically significant different from compared to indicated groups, *p* < 0.05. Error bars represent SEM.

To identify the effects of anti-resorptive drugs on child bone development, we measured femur bone length and analyzed bone mass in the experimental groups using three-dimensional microstructural (μCT) imaging. Injection of the anti-RANKL antibody or ZOL has been reported to cause decreased tibial bone length in adult mice^24–26^. In the present study, there were no significant differences in regard to femur bone length at 8 weeks of age following a single injection or long-term administration of the anti-RANKL antibody in growing mice as compared to the control group. On the other hand, ZOL-treated mice treated that received either a single injection or weekly administration for 7 weeks showed significantly shorter femur bone length in a dose-dependent manner than mice in the saline-injected and anti-RANKL antibody-treated groups (Fig. 2A,D, see Supplementary Fig. S3).

In previous studies, denosumab and bisphosphonates were found to increase bone mass in adult mice^24,26^, as well as human children during development^12,13^. Consistent with those results, femoral bones in both the anti-RANKL antibody- and ZOL-treated mice in the present study showed increased bone mass regardless of the administration period (Fig. 2B,E, see Supplementary Fig. S3). Both the anti-RANKL antibody- and CD-ZOL injected groups showed greater than 100% bone mineral density, thus it could not be accurately measured (data not shown). Tibiae bone histological sections from the anti-RANKL antibody- and ZOL-treated mice were stained with toluidine blue, which revealed increased bone mass in the proximal tibiae metaphysis, as previously described^24,25^. Furthermore, staining with tartrate-resistant acid phosphatase (TRAP), a histochemical marker of osteoclasts, revealed a significantly lower number of osteoclasts in the long-term anti-RANKL antibody-treated mice as compared to the saline-injected and RfD-ZOL-treated groups. In contrast, there was no significant difference regarding the number of tibia osteoclasts in the Ref- and CD-ZOL-treated mice as compared to those given saline (Fig. 2C,F). Bone volume in these mice was high, thus we were unable to perform dynamic histometric analysis.

### CD-ZOL treatment resulted in delayed tooth eruption

**Figure 3:**
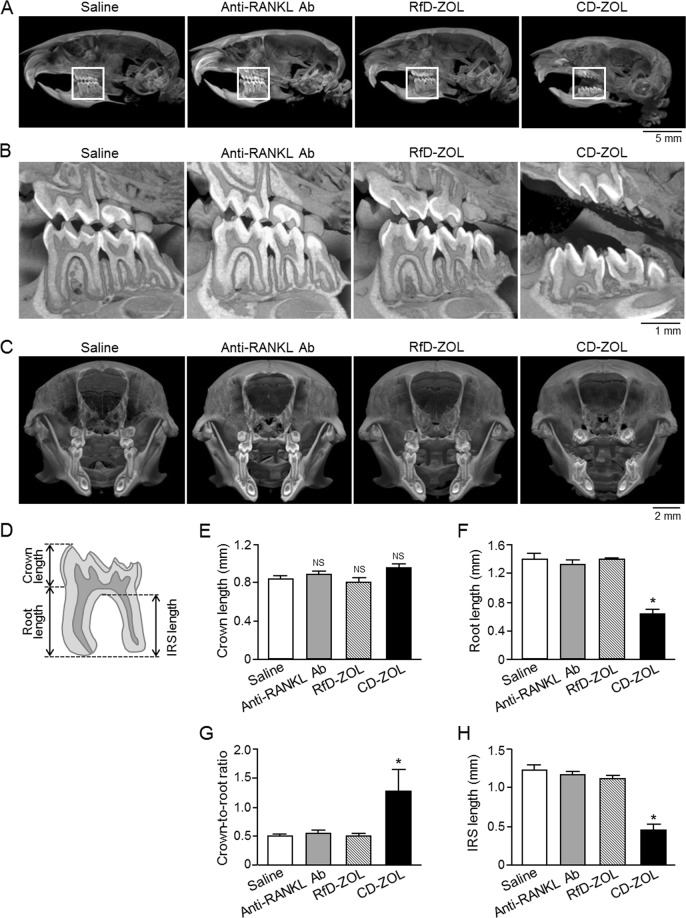
Effects of long-term administration of anti-RANKL antibody or ZOL on cranial bone and tooth development. Micro-computed tomography (μCT) findings of (**A**) sagittal facial bone, (**B**) sagittal left molar, and (**C**) coronal section in 8-week-old mice administered saline, anti-RANKL antibody (Ab) at 2.5 mg/kg, RfD-ZOL at 0.08 mg/kg, or CD-ZOL at 3.0 mg/kg weekly for 7 weeks. (**B**) Higher magnification of white-boxed regions in (**A**). Representative findings are shown in (**A**–**C**). (**D**) Illustration of first molar measurement positions. (**E**) Crown length (between first mesial-buccal crown tip point and cement-enamel junction), (**F**) root length (between cement-enamel junction and lowest root tip point of mesial-buccal root) were measured, and (**G**) crown-to-root ratio and (**H**) interradicular septum (IRS) length (between buccal furcation area and lowest root tip point of mesial-buccal root) of the lower first molar were determined in 8-week-old mice administered saline (n = 6), anti-RANKL Ab (n = 5), RfD-ZOL (n = 6), or CD-ZOL (n = 5). Statistical differences were assessed by one-way ANOVA with Tukey-Kramer’s test. ^*^Statistically significant different from compared to all the other groups, *p* < 0.05. Error bars represent SEM.

Three-dimensional μCT analysis of skulls from 8-week-old mice that received a single injection or long-term treatment of CD-ZOL showed delayed tooth eruption in both of those groups as compared to the saline-injected group, as previously reported^24,25^, whereas the anti-RANKL antibody-treated group demonstrated normal tooth growth (Fig. 3A–C, see Supplementary Fig. S3). Next, we measured crown and root lengths to determine the crown-to-root ratio, and also determined interradicular septum length, and used those values as tooth developing indexes in an attempt to determine the cause of delayed tooth eruption. There were no significant differences between the anti-RANKL antibody- and RfD-ZOL-treated groups as compared to the saline-injected group for tooth index values (Fig. 3E–H). On the other hand, CD-ZOL-treated mice showed normal crown length and decreased root length as compared with the saline-injected and anti-RANKL antibody-treated groups, resulting in a significantly increased crown-to-root ratio (Fig. 3E–G), while the interradicular septum length was also significantly decreased in that group (Fig. 3H).

### Anti-RANKL antibody suppressed osteoclastogenesis in alveolar bone and ZOL significantly suppressed osteoblastogenesis

**Figure 4:**
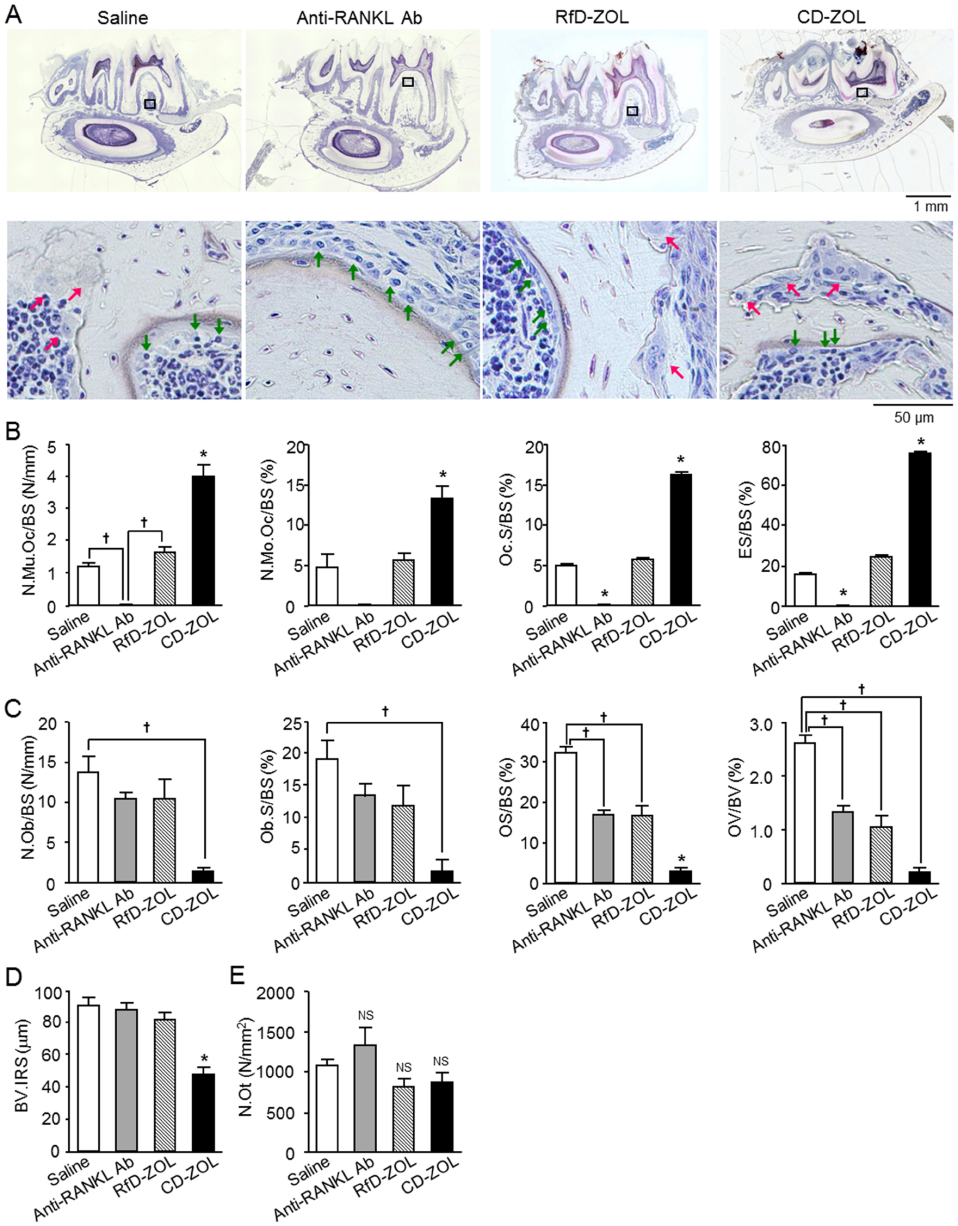
Effects of long-term administrations of anti-RANKL antibody or zoledronic acid on mandibular alveolar bone. (**A**) Villanueva staining of mandibular alveolar bone of 8-week-old mice administered saline, anti-RANKL antibody (Ab) at 2.5 mg/kg, RfD-ZOL at 0.08 mg/kg, or CD-ZOL at 3.0 mg/kg weekly for 7 weeks. Higher magnification images of black-boxed regions in upper panels are shown in lower panels. Green arrows indicate osteoblasts and red arrows osteoclasts. Representative results are shown. (**B**–**E**) Mandibular alveolar bone static parameters were determined using bone morphometric analysis, including (**B**) number of multinucleated osteoclasts (N.Mu.Oc/BS), number of mononuclear osteoclasts (N.Mo.Oc/BS), osteoclast surface (Oc.S/BS), and eroded surface area (ES/BS) on the bone surface, (**C**) number of osteoblasts (N.Ob/BS), osteoblast surface (Ob.S/BS), osteoid surface area (OS/BS) on bone surface, and osteoid volume per bone volume (OV/BV), (**D**) bone volume of interradicular septum (BV.IRS), and (**E**) number of osteocytes (N.Ot). Four mice from each of the saline, anti-RANKL Ab, RfD-ZOL, and CD-ZOL groups were used. Statistical differences were assessed by one-way ANOVA with Tukey-Kramer’s test. Statistically significant different from compared to ^*^all the other groups, or ^†^indicated groups, *p* < 0.05. Error bars represent SEM.

To further investigate the cause of delayed tooth eruption, we performed histomorphometric analysis of alveolar bone samples obtained from mandibular first molar sections, following Villanueva staining^27,28^. In the group treated with the anti-RANKL antibody for 7 weeks, interradicular septum bone volume was increased as compared to the saline-injected group, whereas that was decreased in the CD-ZOL-treated group, because of the shorter root as compared to the saline-injected group (Fig. 4A,D). Mice treated with the anti-RANKL antibody showed no osteoclasts on bone surfaces, which resulted in a lower amount of erosion in that location than in the saline-injected group. In contrast, ZOL treatment increased the numbers of osteoclasts in a dose-dependent manner as well as eroded surface area as compared with the saline-injected and anti-RANKL antibody-treated groups (Fig. 4B), which confirmed previously reported findings^25^. Furthermore, as compared to the control, there was a significant decrease in number of alveolar bone osteoblasts and osteoblast surface on bone surfaces (Ob.S/BS) in the CD-ZOL-treated group, and a tendency for decreased Ob.S/BS in the RfD-ZOL-treated group, while the anti-RANKL antibody-treated group showed no change (Fig. 4C). Also, the osteoid surface of alveolar bone surrounding the lower first molar and osteoid volume/bone volume was significantly decreased in the 3 experimental groups (Fig. 4C), whereas the number of osteocytes was not different (Fig. 4E).

## Discussion

Clinical trials of anti-resorptive drugs for treatment of pediatric bone diseases have been conducted worldwide in recent years^13,29,30^. Among the treatments examined, denosumab has been shown to be a highly effective novel anti-resorptive drug, though basic information regarding its effects in small animals is insufficient, especially during growth stages, as it is a human antibody preparation that has no cross-reactivity in mice. Another effective medication is ZOL, a strong bisphosphonate administered for treatment of bone metastasis in adults. It is anticipated that these major anti-resorptive drugs will be applied for bone diseases in pediatric cases, such as osteogenesis imperfecta^13^, giant cell bone tumor^29^, and osteosarcoma^30^, in the near future.

We examined the effects of mouse anti-RANKL antibody and ZOL administrations in growing mice. Those treated with the antibody showed normal body length and weight, as well as tooth eruption, while those treated with ZOL showed smaller bodies and lower body weight, along with delayed tooth eruption, which occurred in a dose-dependent manner. In human adults, the mean serum half-life of the anti-RANKL antibody is approximately 1 month, which is significantly longer than that of ZOL at approximately 24 hours^31,32^. However, our findings suggested that ZOL, especially when given at a high therapeutic dose, showed longer and sustained action as compared with the anti-RANKL antibody in growing mice, the same effects seen in grown adults. Bisphosphonates have been reported to have a high affinity to hydroxyapatite^7^, thus a prolonged duration of drug action and toxic effects may be exerted in children as compared to anti-RANKL antibody administration. Moreover, high-dose ZOL is known to cause osteoblast inhibition^33^ and cartilage suppression during the administration period^34^, as well as have possible hepatotoxicity effects^35,36^, thus additional investigations from the viewpoint of toxicity of high-dose ZOL are needed to elucidate related growth inhibition mechanisms. RANKL deficiency evokes swelling of the spleen and extramedullary hematopoiesis^23^. As a result, in contrast to a RANKL-deficient mouse phenotype, developed spleens do not show significant differences in growing mice, because RANKL activity in the prenatal stage is adequate for mouse spleen formation^37^.

Another phenotype seen in RANKL-deficient mice is a complete lack of osteoclasts, and these animals develop severe osteopetrosis as well as tooth eruption defects^23^. In the present study, the anti-RANKL antibody significantly decreased osteoclasts and increased bone mass, with an osteopetrotic phenotype shown in long bones of growing mice. The ZOL-injected group also demonstrated a marble bone pattern in long bones, while the number of osteoclasts was nearly equal to that seen in the control. ZOL inactivates osteoclasts^38^ and long-term administration was shown to increase bone mass without decreasing osteoclast number in adult mice^39^, with similar results obtained with the present growing mice. While the anti-RANKL antibody-treated mice showed higher bone volume as compared to RfD-ZOL-treated mice, femur length was not different as compared to control mice. These results suggest that osteoblastic bone formation in developing long bones is induced with or without osteoclasts during treatment with anti-resorptive drugs. Short stature, a phenotype seen in young osteopetrosis patients^40^, is thought to be caused by delayed bone growth and high bone mineral density, though requires further investigation from a perspective other than examination of osteoblasts and osteoclasts.

We considered that the shorter long bone seen in the high-dose CD-ZOL-group was caused by suppressed chondrocyte differentiation, because repeated administration in developing animals during endochondral ossification has been reported to induce retention of the endochondral cartilage matrix within cortical bone and loss of bone length^41^. Continuous ZOL administration in growing mice for a short period was reported to decrease the TRACP5b level in serum and increase bone formation in the metaphysis, while a tendency for decreased tibia length was also noted^34^. Once treatment has finished, development of metaphysis restarts with normal epiphyseal cartilage, while the TRAP level in serum remains low. Following a single injection of high-dose ZOL, the epiphyseal plate and trabeculae remain nearly normal, though not after long-term administration in the present study, could explain with that previous report^34^.

Marks and Cahill placed replica metal teeth in tooth extraction cavities expected to support eruption without normal tooth tissue, and found that the dental follicle is essential for tooth eruption^42^. The coronal half of the dental follicle promotes alveolar bone osteoclastogenesis, which is mediated by RANKL, and creates an eruption pathway with continuous osteoclastic bone resorption of alveolar bone near the tooth crown^43–46^. The present anti-RANKL antibody-treated mice showed normal tooth development with a reduced number of osteoclasts. On postnatal day 5, a major burst of osteoclast differentiation typically occurs in mice^47^ and our findings suggest that the eruption pathway is created by osteoclasts early in the postnatal period. In contrast, even a single injection of high-dose ZOL on postnatal day 7 decreased tooth eruption in the present mice, which has also been observed in human studies^48,49^. As preparation for tooth eruption, the basal half of the dental follicle promotes osteogenesis and creates a trabecular morphology in alveolar bone^45^. In human molars, interradicular septum growth is especially important for tooth eruption^50^. Therefore, increased alveolar bone volume and length induced by osteoblasts from the dental follicle are thought to push teeth and accelerate eruption. In the present study, ZOL reduced the number of osteoblasts in the interradicular septum of first molars, which resulted in eruption delay. Our results suggest that decreased osteogenesis derived from the dental follicle by administration of ZOL delays tooth eruption. In rodents, lower molar development is initiated in the embryo and continues after birth^21^, while maximal bone formation for development begins from postnatal day 9–14^51^. Based on the present findings, we consider that ZOL might crucially affect alveolar bone formation during the period of lower first molar development.

Tooth root formation in CD-ZOL administrated mice was substantially decreased, while the anti-RANKL antibody group showed normal root length, thus it is considered that osteoclasts are not essential for root formation. Takahashi *et al*. reported that dental follicle mesenchymal progenitor cells differentiated into cementoblasts on the acellular cementum, as well as periodontal ligament cells and alveolar cryptal bone osteoblasts during root formation^52^. Deletion of parathyroid hormone signaling by dental follicle cells induces loss of periodontal attachment and failure of tooth eruption along with up-regulation of some matrix proteins. In the present study, CD-ZOL-injection significantly decreased the number of osteoblasts in alveolar bone. The lower first molar root in mice is known to be initiated on postnatal day 6 to 8^53^, while ZOL administration likely decreases the number of cementoblasts and periodontal ligament cells, leading to failure of tooth eruption. The present ZOL-treated group showed both maxilla and mandible tooth eruption failure, resulting in open bite in the occlusion.

Contrary to other well-known ZOL effects, mono/multi-osteoclast numbers were increased, resulting in a high percentage of teeth with an eroded surface in mice in the long-term ZOL-treated group. Growing alveolar bone has a high mount of turnover^54^, with bone formation and bone resorption enhanced by osteoblast/osteoclast coupling. ZOL treatment suppressed osteoclast function, though bone resorptive activity was partially retained. Activated resorption in growing alveolar bone without adequate formation might have ostensibly increased the ES/BS ratio in the ZOL-treated group as compared to the control. In some cases, bone disease is accompanied by tooth or jaw bone abnormalities, such as osteogenesis imperfecta accompanied by dentinogenesis imperfecta. Additional research is needed to clarify the effects of anti-resorptive drugs on teeth of pediatric patients affected by bone disease.

In summary, administration of anti-resorptive drugs resulted in increased bone volume in developing mice. Furthermore, the bisphosphonate ZOL induced growth retardation, while the anti-RANKL antibody showed no developmental side-effects. Inhibition of osteogenesis in the dental follicle by ZOL led to delayed tooth eruption, though normal tooth eruption occurred, in contrast to inhibition of osteoclastogenesis by the anti-RANKL antibody. These results are important in regard to determination of appropriate treatment protocols for affected child patients. When an anti-resorptive drug is given for treatment of pediatric disease, both the concentration and administration period must be carefully determined.

## Methods

### Reagents

A rat anti-mouse RANKL antibody (clone OYC1; Oriental Yeast, Tokyo, Japan) was used in this study. ZOL (Zometa; Novartis Pharma, Tokyo, Japan), saline (Otsuka Pharmaceutical, Tokyo, Japan), and rat IgG (Medical & Biological Laboratories, Nagoya, Japan) were obtained from their respective commercial sources.

### Animals and breeding environment

Newborn C57BL/6J mice, born to inbred parents over 8 weeks old (Sankyo Labo Service Corporation, Inc., Tokyo, Japan), were used in these experiments, as the C57BL/6J strain has been widely used for anti-resorptive drug experiments^26^. Eight or 9 pups, breastfed by a single mother, were weaned at 4 weeks, then fed with powdered chow for 1 week and normally thereafter. The experiments were started with 1-week-old healthy male mice with weights ranging from 3.0–5.6 g (average 4.3 g). The animals were housed under standard laboratory conditions and euthanized at 8 weeks of age. All experiments were performed in full compliance with the Guidelines for Animal Experiments of Showa University, Showa University Animal Care and Use Committee, Japan, after receiving approval from the committee (certificate numbers 17050 and 18073).

### Single injection of anti-resorptive agents

The anti-resorptive drug concentrations, 2.5 mg/kg of anti-mouse RANKL antibody and 0.08 mg/kg ZOL (RfD-ZOL), were determined based on the adult therapeutic dose and calculated based on body weight. The high dosage of ZOL (CD-ZOL), 3.0 mg/kg/week for 7 weeks (total 21 mg/kg), was decided based on the cumulative dose for human child osteogenesis imperfecta patients (mean dose 29 mg/kg), as previously described^48^.

Thirty-three 1-week-old male mice were randomly assigned to 5 different groups, as follows: saline (n = 4), rat IgG (n = 8), anti-RANKL antibody (n = 8), RfD-ZOL (n = 5), and CD-ZOL (n = 8). Drugs were subcutaneously administrated to the 1-week-old mice with a single injection using a 27G, 1.5-inch needle (Terumo Corporation, Tokyo, Japan). Findings from 4 mice in the saline, 5 in the rat IgG, 6 in the anti-RANKL antibody, 5 in the RfD-ZOL, and 7 in the CD-ZOL groups were analyzed, as 3 in the rat IgG-treated, 2 in the anti-RANKL-treated, and 1 in the CD-ZOL-treated groups died during the experimental period.

### Long-term anti-resorptive drug treatment protocol

Twenty-eight 1-week-old male mice were randomly assigned to 5 different groups, as follows: saline (n = 6), rat IgG (n = 5), anti-RANKL antibody (n = 5), RfD-ZOL (n = 6), and CD-ZOL (n = 6). The first administration of each drug was at the age of 1 week and then continued once a week for a total of 7 injections. Findings from 6 mice in the saline, 5 in the rat IgG, 5 in the anti-RANKL antibody, 6 in the RfD-ZOL, and 5 in the CD-ZOL groups were analyzed, as 1 in the CD-ZOL-treated group died during the experimental period.

### Determination of growth index and percentage spleen weight

Determination of body length, measured from the nasal tip to anus (naso-anal length), was performed at 8 weeks of age, while body weight was determined weekly until 8 weeks and percentage spleen weight was calculated using spleen weight/body weight at 8 weeks of age.

### Micro-computed tomography analysis

The head, mandibular alveolar molar bone, and right femur were obtained from mice at the age of 8 weeks, then fixed in 70% ethanol and scanned using a ScanXmate-L090H (Comscantecno, Yokohama, Japan). For µCT measurements, the samples were analyzed at 80 kV and 81 µA. Three-dimensional microstructural image data were reconstructed and analyzed using a TRI/3D-BON-FCS system (Ratoc System Engineering, Tokyo, Japan). Bone volume/tissue volume (BV/TV) and other bone morphometric analyses of the femur at approximately 1.0 mm above the distal growth plate were performed as previously described^55^.

### Histological analysis

The left tibia was obtained at the age of 8 weeks and dehydrated in 70% ethanol, then embedded in glycol methacrylate and longitudinally sectioned into 3-μm slices using a microtome. The sections were stained with toluidine blue or tartrate-resistant acid phosphatase (TRAP, histochemical marker of osteoclasts), as previously described^56,57^. Static histomorphometry evaluations of first molar alveolar bone samples were done as previously described^27,58^. Briefly, the right mandibular was obtained at 8 weeks and fixed in 70% ethanol, then stained for 4 days using the Villanueva method^27,28^. Subsequently, bone samples were dehydrated, embedded in methyl methacrylate, and sectioned in a sagittal manner into 5-μm slices using a microtome. Osteoclast counts were performed by an experienced researcher using polarization microscopy. Static parameters for bone formation and resorption were determined in an area 1.0 mm longitudinal and 0.75 cm horizontal from the interradicular septum by direct tracing using a Histometry RT digitizer (System Supply, Yokohama, Japan), then computed using specialized software (CSS-840 cancellous bone morphometry version; System Supply). For static parameters, osteoclast numbers/bone surface (N.Oc/BS), osteoblast numbers/bone surface (N.Ob/BS), and osteocyte numbers (N/Ot) were counted, while for structural parameters osteoclast surface (Oc.S/BS), eroded surface area (ES/BS), osteoblast surface (Ob.S/BS), osteoid surface area (OS/BS) on the bone surface, osteoid volume/bone volume (OV/BV), and bone volume of the interradicular septum (BV.IRS) were determined.

### Statistical analysis

Results were analyzed using Student’s-t test or one-way ANOVA with Tukey-Kramer’s test. All values are expressed as the mean ± SEM. A P value < 0.05 was considered to indicate a significant difference.

## Supplementary information

Supplementary Figures
