# Supplementary material for: Biological Effects of Anti-RANKL Antibody and Zoledronic Acid on Growth and Tooth Eruption in Growing Mice
Source: Sci Rep. 2019 Dec 27;9:19895. doi: 10.1038/s41598-019-56151-1 (PMC6934544; doi:10.1038/s41598-019-56151-1)
Supplement: Supplementary file 1 — Supplementary Figures [file 41598_2019_56151_MOESM1_ESM.docx]

**Biological Effects of Anti-RANKL Antibody and Zoledronic Acid on Growth and Tooth Eruption in Growing Mice**

Running title: Effects of Anti-resorptive Drugs on Tooth Eruption

Motoki Isawa, Akiko Karakawa, Nobuhiro Sakai, Saki Nishina, Miku Kuritani, Masahiro Chatani, Takako Negishi-Koga, Masashi Sato, Mitsuko Inoue, Yukie Shimada, Masamichi Takami*

**Supplementary Figures**

**Supplementary Figure 1**


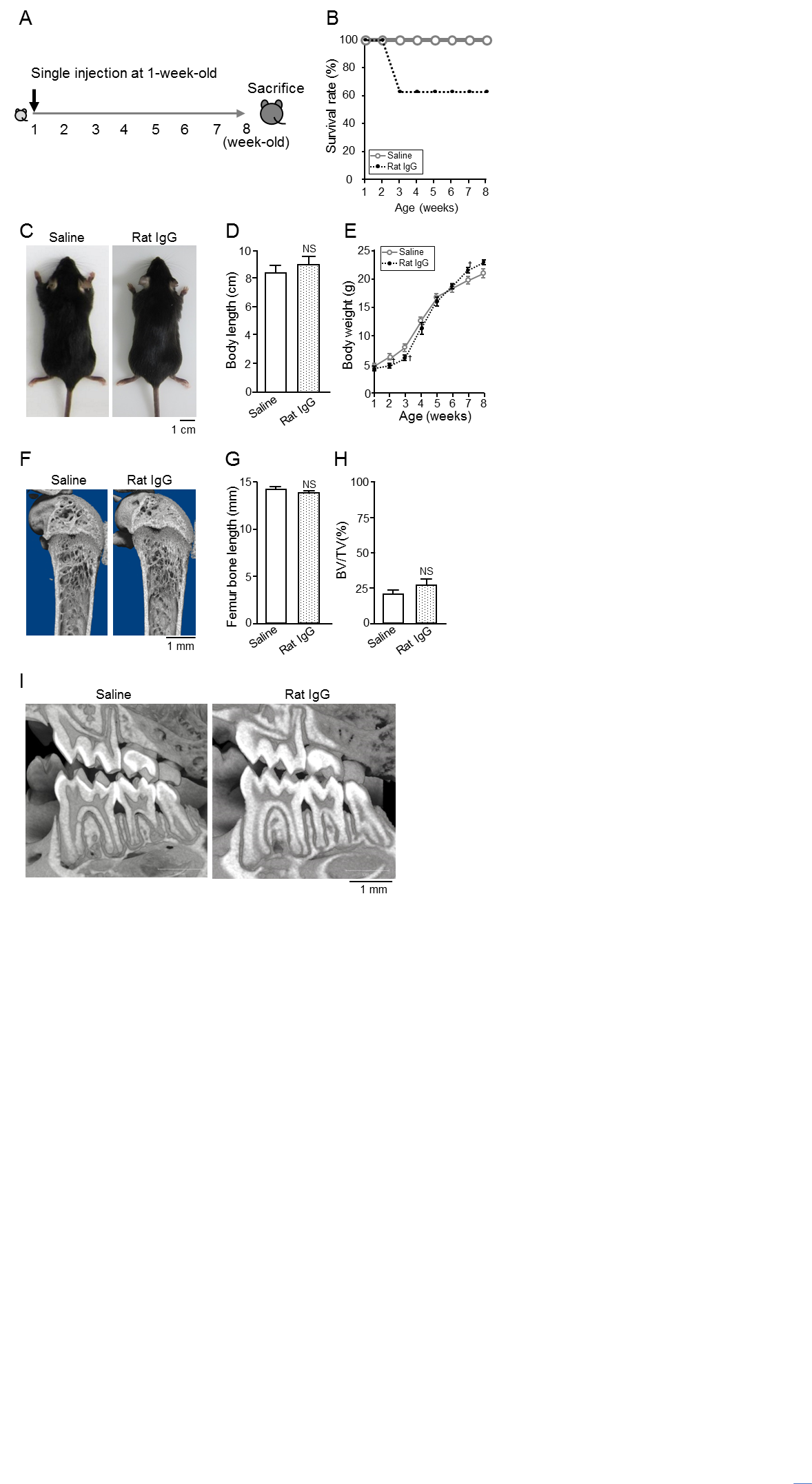


Effects of single injection of rat IgG on mouse growth. (A) Single injection protocol. (B) Survival rates after single injection at 1 week of age in saline (control, n=4) and 2.5 mg/kg of rat IgG (negative isotype control, n=8) groups. At 8 weeks of age, the survival rates of mice in those groups were 100% and 63%, respectively. (C) Growth appearance, (D) naso-anal length, (E) weight curves, (F) distal femur appearance, (G) femur length, (H) bone volume/tissue volume (BV/TV) ratio, and (I) μCT findings of sagittal left molars of 8-week-old mice after single injection of saline (n=4) or rat IgG (n=5). Statistical differences were assessed by Student’s-t test. ^†^Statistically significant different from compared to indicated groups, *p* <0.05. NS, not significant. Error bars represent SEM.

**Supplementary Figure 2**

**
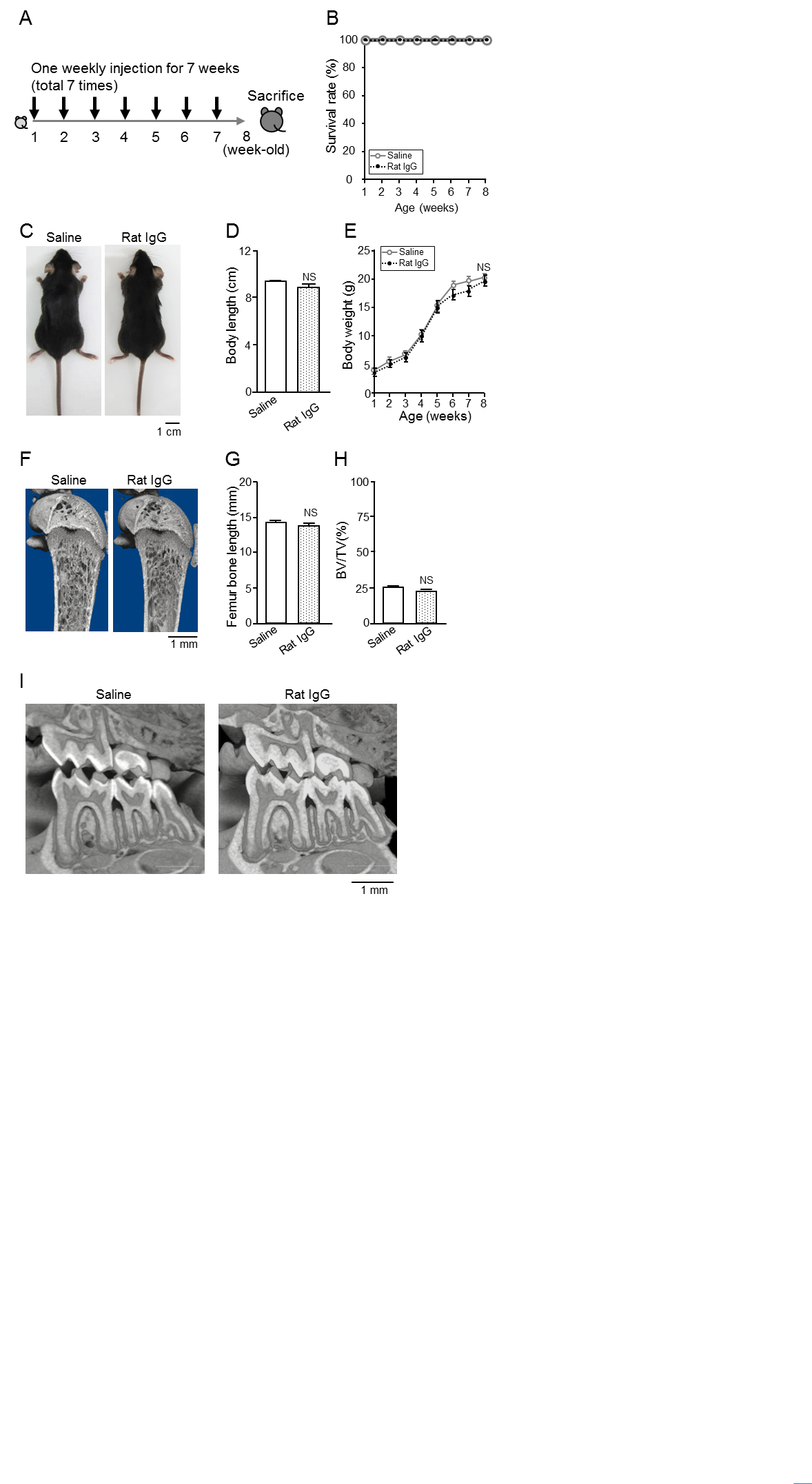
**

Effects of long-term administration of rat IgG on mouse growth. (A) Long-term administration protocol. (B) Survival rates after once-weekly administration of saline (control, n=6) or 2.5 mg/kg of rat IgG (negative isotype control, n=5) for 7 weeks. At 8 weeks of age, the survival rate of mice both groups was 100%. (C) Growth appearance, (D) naso-anal length, (E) weight curves, (F) distal femur appearance, (G) femur length, (H) bone volume/tissue volume (BV/TV) ratio, and (I) μCT findings of sagittal left molars of 8-week-old mice after long-term administrations of saline (n=4) or rat IgG (n=5). Statistical differences were assessed by Student’s-t test. NS, not significant. Error bars represent SEM.

**
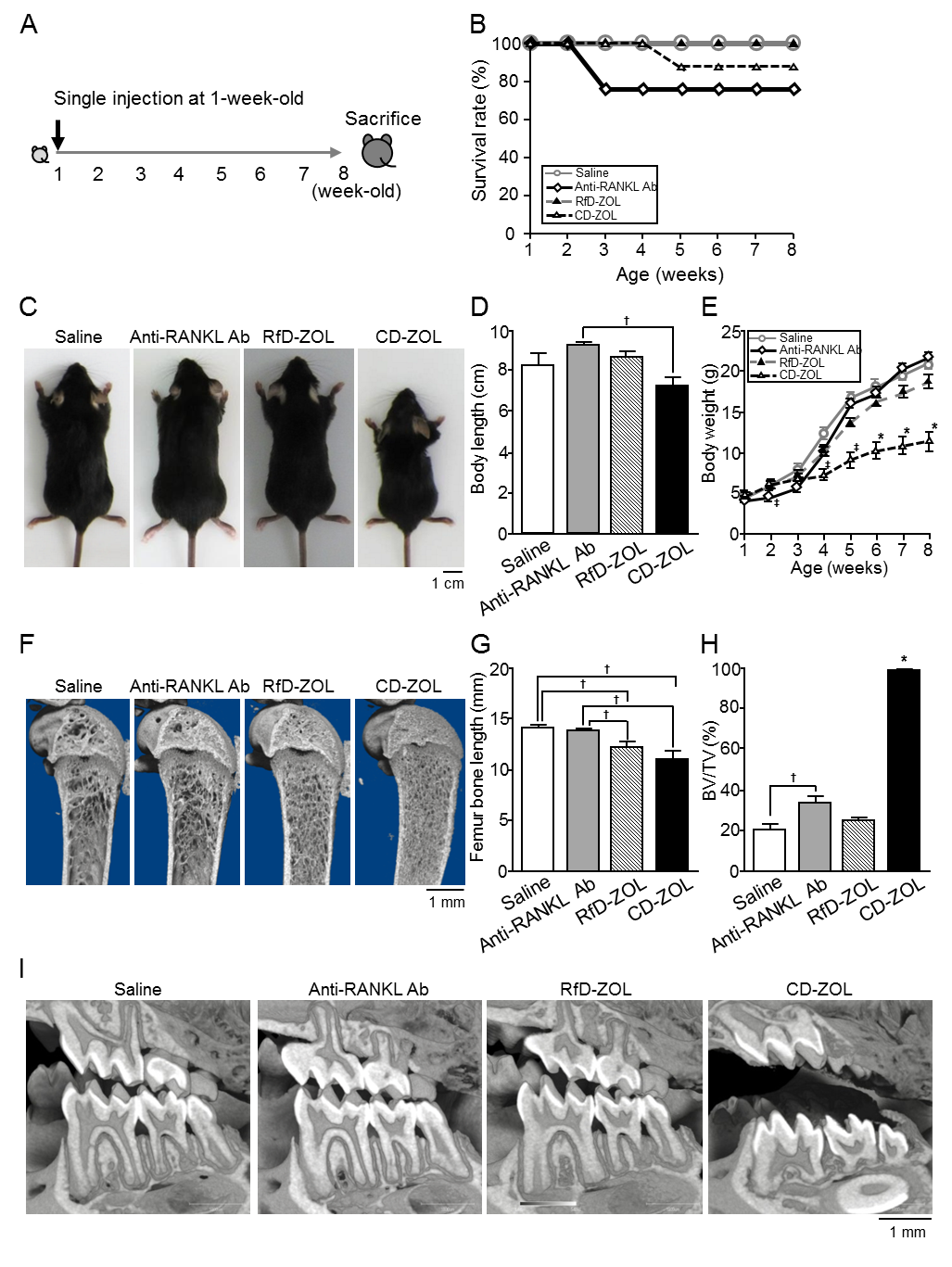
Supplementary Figure 3**

Effects of single injection of anti-RANKL antibody or ZOL on mouse growth. (A) Single injection protocol. (B) Survival rates after single injection at 1 week of age in saline (control, n=4), anti-RANKL antibody (Ab) at 2.5 mg/kg (n=8), RfD (reference dose)-ZOL at 0.08 mg/kg (n=5), and CD (cumulative dose)-ZOL at 3.0 mg/kg (n=8) groups. At 8 weeks of age, the survival rates of mice in those groups were 100%, 75%, 100%, and 88%, respectively. (C) Growth appearance, (D) naso-anal length, (E) weight curves, (F) distal femur appearance, (G) femur length, (H) bone volume/tissue volume (BV/TV) ratio, and (I) μCT findings of sagittal left molars of 8-week-old mice after single injection of saline (n=4), anti-RANKL Ab (n=6), RfD-ZOL (n=5), or CD-ZOL (n=7). Statistical differences were assessed by one-way ANOVA with Tukey-Kramer’s test. Statistically significant different from compared to ^*^all the other groups, ^†^indicated groups, or ^‡^saline, *p* <0.05. Error bars represent SEM.

**Supplementary Figure 4**

**
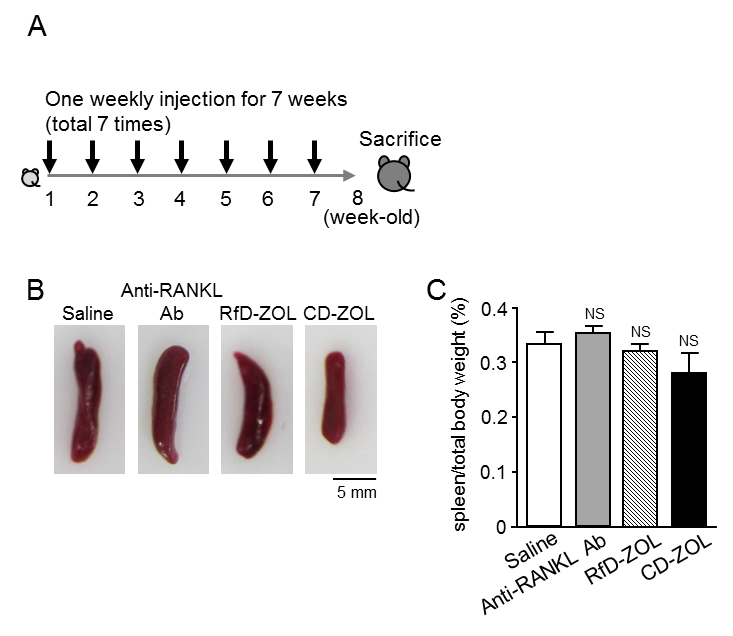
**

Effects of long-term administration of anti-RANKL antibody or ZOL on spleen. (A) Long-term administration protocol. (B) Spleen appearance and (C) ratio of spleen/total body weight were determined in 8-week-old mice administered saline (n=6), anti-RANKL Ab (n=5), RfD-ZOL (n=6), or CD-ZOL (n=5). Statistical differences were assessed by one-way ANOVA with Tukey-Kramer’s test. NS, not significant. Error bars represent SEM.
